# Optimal-Reference Excited State Methods: Static Correlation at Polynomial Cost with Single-Reference Coupled-Cluster Approaches

**DOI:** 10.1021/acs.jctc.5c00172

**Published:** 2025-04-01

**Authors:** Sylvia J. Bintrim, Kevin Carter-Fenk

**Affiliations:** Department of Chemistry, University of Pittsburgh, Pittsburgh, Pennsylvania 15218, United States

## Abstract

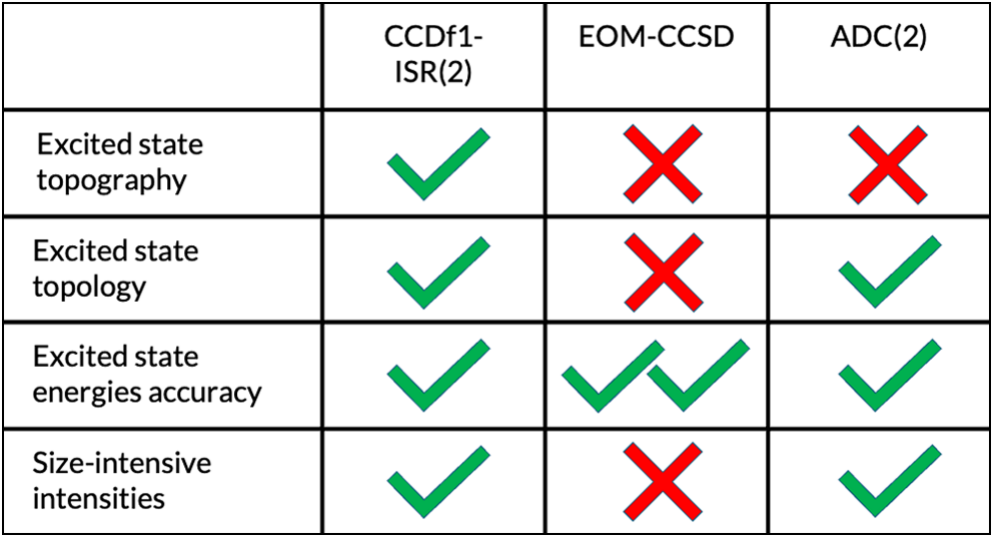

Accurate yet efficient modeling of chemical systems with pronounced static correlation in their excited states remains a significant challenge in quantum chemistry, as most electronic structure methods that can adequately capture static correlation scale factorially with system size. Researchers are often left with no option but to use more affordable methods that may lack the accuracy required to model critical processes in photochemistry such as photolysis, photocatalysis, and nonadiabatic relaxation. A great deal of work has been dedicated to refining single-reference descriptions of static correlation in the ground state via “addition-by-subtraction” coupled cluster methods such as pair coupled cluster with double substitutions (pCCD), singlet-paired CCD (CCD0), triplet-paired CCD (CCD1), and CCD with frozen singlet- or triplet-paired amplitudes (CCDf0/CCDf1). By combining wave functions derived from these methods with the intermediate state representation (ISR), we gain insights into the extensibility of single-reference coupled cluster theory’s coverage of static correlation to the excited state problem. Our CCDf1-ISR(2) approach is robust in the face of static correlation and provides enough dynamical correlation to accurately predict excitation energies to within about 0.2 eV in small organic molecules. We also highlight distinct advantages of the Hermitian ISR construction, such as the avoidance of pathological failures of equation-of-motion methods for excited state potential energy surface topology. Our results prompt us to continue exploring optimal single-reference theories (excited state approaches that leverage dependence on the initial reference wave function) as a potentially economical approach to the excited state static correlation problem.

## 1 Introduction

Electron correlation beyond Hartree–Fock theory is the central problem in quantum chemistry. While the distinction is somewhat arbitrary, it is often conceptually useful to partition the correlation energy into dynamical and nondynamical (static) correlation effects.^1−11^ Of the two, dynamical correlation is simpler to incorporate into theoretical model chemistries as it manifests from spontaneous repulsions between pairs of electrons that can be incorporated into a single-reference formalism. On the other hand, static correlation results from many possible ground state reference determinants being of roughly equal energy and importance, and its affordable incorporation into practical calculations remains one of the grand challenges of electronic structure theory.

Density functional theory (DFT) captures electron correlation at mean-field cost, but the included correlation is almost exclusively dynamical, and the Hohenburg–Kohn and Kohn–Sham theorems preclude any obvious extension of DFT to the multideterminant case,^12,13^ though there are efforts in this direction.^14−18^ Beyond the challenges posed by defining a static correlation functional under the constraints of a single, spin-pure determinant, DFT methods are also afflicted by self-interaction errors^19−21^ that cause a myriad of problems including underestimated barrier heights,^22,23^ spuriously low-energy charge-transfer excitations,^24−35^ and fundamental difficulties with local approximations innate to many-body expansion algorithms.^36^

Unlike DFT, wave function theories (WFTs) more naturally lend themselves to a depiction of static correlation as electron self-interactions can be exactly eliminated.^37^ However, WFT methods that can treat static correlation affordably are few in number, generally scaling factorially with the number of correlated orbitals in the system. Some notable examples are complete active space (CAS) approaches wherein the full configuration interaction (FCI) coefficients are optimized, either alongside the molecular orbitals (MOs) in a CAS self-consistent field (CASSCF) procedure or without MO optimization (CASCI).^38−41^ While the extension to multireference situations remains somewhat formally ambiguous, multireference coupled-cluster (CC) theories are also a hotbed of active methodological development.^42−44^ Furthermore, cumulant functional methods such as density matrix functional^45−50^ and natural orbital functional^51−58^ theories alone, or interfaced with WFT methods like Møller–Plesset perturbation theory,^59,60^ have also shown promise in economical descriptions of static correlation.

The discussion heretofore has been dedicated exclusively to electron correlation in the ground state because most efforts toward describing static correlation have focused on improving the ground state. When facing static correlation problems in excited state calculations, the computational chemist’s affordable options are sorely lacking.^61^ This deficiency in suitable options can be problematic when modeling photolysis or nonadiabatic dynamics more generally. For example, single-reference methods often overestimate nonradiative decay yields in surface-hopping simulations.^62^ Available multireference methods include CASCI, state-specific CASSCF,^63−67^ state-specific CI,^68^ multiconfigurational linear response atop a CASSCF reference,^69−71^ excited state mean-field theory,^72−77^ and multireference algebraic diagrammatic construction (MR-ADC).^78−82^ Apart from excited state mean-field theory, all of the aforementioned approaches use a CASSCF reference state that requires a bespoke selection of active orbitals, leading to varying results depending on active space selection. In addition, the factorial scaling with respect to active space size often limits applications of CASSCF-reference theories to a subset of explicitly correlated orbitals, possibly resulting in the omission of important correlation effects. Selecting an active space guided by chemical intuition alone amplifies this risk, but the density matrix renormalization group (DMRG) can make larger active space sizes more accessible by application of singular value decomposition to compress the variational wave function, and information-theoretic metrics can be used to automatically select an active space.^83−85^

Among the single-reference approaches that are capable of accounting for some static correlation are spin-flip variants of ADC,^86−88^ equation-of-motion CC (EOM-CC),^89−91^ and time-dependent DFT (TD-DFT),^92,93^ albeit at the cost of forgoing spin-pure excited states. There has also been a recent surge of interest in seniority-zero CC approaches such as the pair coupled cluster doubles (pCCD)^94−98^ theory (described below) with extensions to excited states including EOM-pCCD, linear response pCCD, and orbital-optimized pCCD for doubly excited states.^99−103^ However, as we will later discuss, pCCD is not invariant to unitary transformations of the occupied or virtual orbitals. After orbital optimization or localization, the pCCD orbitals are a well-defined basis for the pCCD energy, but the lack of orbital invariance of the energy may hinder extensions to local correlation theories or fragment-based approaches and may have a detrimental impact on excited state analyses.^104^ Excited state properties may be adversely affected by the fact that orbital-optimized pCCD introduces spurious spatial symmetry breaking, which in concert with the orbital dependence of pCCD, may introduce artifacts in the spectra of small, symmetric molecules such as broken degeneracies and incorrect selection rules. Despite the lack of orbital invariance, pCCD-based methods have found success in applications from vertical excitation energies in actinide- and lanthanide-containing complexes to modeling the structure of large organic molecules by interfacing pCCD with embedding methods.^105−108^

In this work, we present a polynomial-scaling, black-box, single-reference, size-consistent excited state approach that is robust in cases of static correlation, provides spin-pure excited states, and employs a Hermitian approach necessary for the description of excited state potential energy surface topology^109,110^ and for the size-intensivity of predicted oscillator strengths. In particular, we will leverage the sensitivity of perturbative excited state approaches based on the intermediate state representation (ISR)^111−115^ to the initial reference wave function, replacing the usual second-order Møller–Plesset (MP2) reference wave function with the first-order approximation to a CC wave function that captures some static correlation effects. This approach was originally introduced by Dreuw and co-workers using CC with double substitutions (CCD) or CC with single and double substitutions (CCSD) but has yet to be thoroughly explored for other CC *ansatze*.^113,116,117^ Namely, “addition-by-subtraction” approximations wherein only certain double substitutions are retained (i.e., the aforementioned pCCD, among others discussed below) have not been assessed in the ISR context. Such approaches might be better suited for systems in which static correlation is more pronounced, thereby making the best of the first-order CC wave function approximation within the ISR.^98^

At the heart of this work is the need to provide computational chemists with more affordable tools that can describe the many incidences in computational photochemistry where static correlation becomes important, such as photolysis and the rich and useful photochemistry of transition metal complexes.^118^ The crux of our foray into such optimal single-reference ISR approaches is the question, “Does the robustness of addition-by-subtraction CC theory to static correlation translate to excited states?” Herein we demonstrate the potential power of polynomial-scaling ISR approaches for capturing a wide array of crucial properties in the photochemistry of statically correlated systems without compromising the accuracy of ISR methods in weakly correlated systems where the performance of ADC is already satisfactory.

## 2 Theory

### 2.1 Addition-by-Subtraction CCD

The standard CCD approach employs an exponential *ansatz* to the wave function
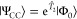
1where |Φ_0_⟩ is usually the Hartree–Fock ground state reference determinant,
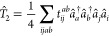
2is the usual double-substitution operator, and *â*_*i*_ and *â*_*a*_^†^ are particle annihilation and particle creation operators, respectively. Throughout this work, occupied orbitals will be indexed as {*i*, *j*, *k*, *l*, ...} and virtual orbitals as {*a*, *b*, *c*, *d*, ...}. The corresponding energy and amplitude equations for CCD are
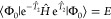
3a
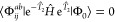
3b

Other authors have shown quite convincingly that judicious removal of some *T̂*_2_ amplitudes can greatly improve the qualitative behavior of CCD when static correlation becomes more important.^98,119−122^ The most aggressive such approximation is known as pCCD, wherein all but the diagonal components of *T̂*_2_ are removed, resulting in,
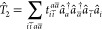
4where barred indices correspond to β-spin orbitals. The pCCD approach is equivalent to a generalized valence bond method known as the antisymmetric product of 1-reference orbital geminals and describes single-and double-bond breaking quite well.^123−129^ Combined with its  computational complexity (after one  transformation from the atomic orbital to molecular orbital basis), the robustness of pCCD in the face of bond breaking has driven considerable interest despite the fact that pCCD lacks invariance to orbital rotations within the occupied or virtual subspaces.

Orbital invariant approaches that retain only the singlet (CCD0) or triplet (CCD1) amplitudes have shown similar success to pCCD in the description of bond breaking, albeit at an increased cost of .^121,130^ Singlet-paired CCD0 substitutions take the form
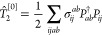
5where
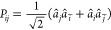
6with the corresponding definition for the singlet-paired creation operator, *P*_*ab*_^†^. The singlet amplitudes are related to the usual CCD *t*_*ij*_^*ab*^ by
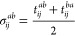
7Likewise, triplet-paired CCD1 takes the form
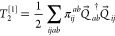
8The product between vector operators *Q⃗*_*ab*_^†^·*Q⃗*_*ij*_ is evaluated as
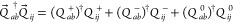
9where their components are (again showing only the annihilation operators explicitly)
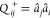
10a
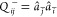
10b
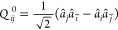
10cFinally, the π_*ij*_^*ab*^ amplitudes are related to the usual CCD amplitudes by
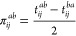
11These singlet- and triplet-paired amplitudes are uncoupled, resulting in an unsatisfactory recovery of dynamical correlation in CCD0 and CCD1. In this work, we will explore the uncoupled CCD0 and CCD1 reference wave functions alongside the FpiCCD approach where the π amplitudes are computed, then frozen and inserted into the full CCD equations.^130^ Hereafter we call this recoupling approach CCDf1, where “f1” means that the triplet amplitudes are frozen. We refer to the analogous method where the singlet amplitudes are computed first and then frozen as CCDf0.

Both CCDf1 and CCDf0 can be viewed as infinite-order solutions to external CC perturbation theory (xCCPT) equations, where *T̂* = *T̂*_*x*_ + δ*T̂* are the full *T̂* amplitudes: a set of external (or frozen) *T̂*_*x*_ and a perturbation δ*T̂*.^131^ In the case of CCDf1, we take *T̂* = *T̂*_2_, and *T̂*_*x*_ = *T̂*_2_^[1]^ are the frozen triplet amplitudes. The wave function can be written as
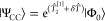
12resulting in the Schrödinger equation
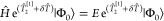
13Premultiplying by e^–*T̂*_2_^[1]^^ gives
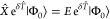
14where *X̂* = e^–*T̂*_2_^[1]^^*Ĥ* e^*T̂*_2_^[1]^^ is the once-similarity-transformed Hamiltonian. This equation can be solved perturbatively, or (in the case of CCDf1 and CCDf0) exactly for δ*T̂* and δ*E*. In the latter case, we note that this corresponds to an infinite-order solution to the external CC equations.

### 2.2 Intermediate State Representation

Within the ISR approach, excitation energies are obtained by solving the secular equation of a shifted Hamiltonian *Ĥ* – *E*_0_,
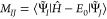
15which presents as a Hermitian eigenvalue problem,
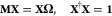
16Key to the ISR approach is the correlated excited state basis,
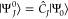
17where,
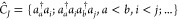
18are physical excitation operators that generate the excited state configuration *J*. The correlated excited states {Ψ_*J*_^0^} are then orthogonalized via the Gram–Schmidt procedure to all intermediate states of lower excitation classes and then orthonormalized among themselves to generate the intermediate states {Ψ̃_*J*_}.

The ISR procedure produces excited states that are properly orthogonal to one another and to the reference state, and the matrix **M** is Hermitian by construction, making ISR a convenient formalism to obtain excited states and size-intensive properties. Notably, the ISR formalism expands the solutions to eq 15 order-by-order both in the fluctuation potential and in terms of the physical excitation operators. For example, first-order in the fluctuation potential corresponds to single excitations only, while second-order in the fluctuation potential results in a second-order treatment of the single excitations with a zero-order treatment of double excitations, and so on.

### 2.3 CC-ISR(2) Approximations

As with ground state perturbation theory, second-order ISR [ISR(2)] energies are obtained using first-order wave functions of the form
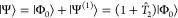
19Dreuw and co-workers noted that, while not strictly formally justifiable, the first-order Taylor-expanded coupled cluster wave function can be written as^113^
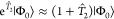
20and inserted directly into the second-order ISR(2) procedure to obtain an approach that they called CCD-ISR(2). The CCD-ISR(2) equations (eq 36 in ref (113)) differ from the simplified ADC(2) equation because the CC *t* amplitudes differ from those of MP2 (the typical choice for the ADC(2) ground state). In preliminary studies of the CC-ADC(2) *ansatz* (the direct use of the simplified ADC(2) expression with CC amplitudes), it was shown that CC amplitudes can improve singlet-to-triplet excitation energies and may be more robust against the divergences that plague the MP2 reference.^116,117^

Although the first-order approximation to the CCD reference wave function is generally stable in bond-breaking problems, CCD struggles to dissociate multiple bonds and may perform poorly in systems where static correlation is important.^121^ Inspired by the relatively positive results obtained using CC-ISR(2), we are interested in further exploring the potential scope of Hermitian excited state methods that result from the ISR construction. In this work, we will explore the use of addition-by-subtraction CC amplitudes within the CC-ISR(2) framework. Specifically, we will insert pCCD, CCD0/1, and CCDf0/1 amplitudes into eq 19 to explore whether the qualitatively good behavior of these approaches for statically correlated ground states are translatable into the excited state manifold via ISR(2).

### 2.4 Brueckner Orbitals

Brueckner orbitals result from rotating the HF determinant to a basis in which the CC *t*_1_ amplitudes are all zero. In the Brueckner orbital basis, there is some account of orbital relaxation through inclusion of *T̂*_1_ substitutions by nature of Thouless’ Theorem.^132^ One consequence of including additional relaxation effects is that Brueckner orbitals often preserve spatial symmetry, even when the HF determinant breaks it.^133^

By setting *t*_1_ to 0 in the CCSD equations, we obtain the occupied-virtual block of the Brueckner modified Fock matrix:
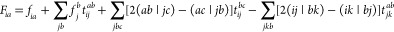
21in terms of spatial orbitals. Since the mean field self-consistency condition is *F*_*ia*_ = 0, we can use existing SCF machinery to successively diagonalize the redefined Fock matrix to obtain a set of Brueckner orbitals. Alternatively, one can directly apply rotations via e^*T̂*_1_^ to the MO coefficients until *t*_1_ = 0 and then semicanonicalize the orbitals. We take the latter approach in this work.

The case of Brueckner pCCD (pBCCD) is notably different due to the lack of orbital invariance of the pCCD energy. Specifically, pBCCD is often accompanied by successive orbital localizations between Brueckner cycles to impart orbital rotations that more significantly incorporate relaxation into the MO basis. Furthermore, Brueckner orbitals without localization do not provide a unique orbital pairing scheme for pCCD.^94^ However, orbital localization results in artificial spatial symmetry breaking that breaks important degeneracies. Because pCCD is sensitive to these changes, localized orbitals may make pCCD excited states difficult to assign and influence selection rules that are leveraged by spectroscopists to inform on *physical* symmetry-breaking in the active sites of metalloenzymes, for instance.^134^ Noting that linear-response pCCD oscillator strengths have been studied alongside the impacts of the choice of orbital basis,^103^ we leave detailed study of pCCD-ISR(2) oscillator strengths for future work. For all of the aforementioned reasons, we chose to use semicanonical pBCCD/pBCCD-ISR(2) without successive orbital localization.

In some sense, Brueckner CCD (BCCD)-ISR(2) is more justifiable than CCD-ISR(2), as the *t*_1_ amplitudes are zero, akin to the usual MP2/ADC(2) case. This formally decouples single excitations from the reference determinant in the CI-like ISR(2) Hamiltonian, whereas CCD-ISR(2) methods simply neglect this coupling. Formally speaking, methods such as CCD- and CCSD-ISR(2) have nonzero *t*_1_ contributions that should impact the singles block of the ISR matrix at all orders in perturbation theory, but these contributions are typically ignored. Herein, we compare the quality of ISR(2) results derived from CC and BCC ground states by neglecting the *t*_1_ contribution to ISR in the first (usual) case and formally decoupling the reference state from the single excitations in the latter.

## 3 Computational Details

The potential energy surfaces of N_2_ were calculated using CASSCF with second-order *N*-electron valence state perturbation theory (CASSCF(6,6)@NEVPT2) using 6 active electrons and 6 active orbitals in the aug-cc-pVTZ basis set,^135^ with resolution of the identity and chain of spheres exchange^136^ for electron-repulsion integrals. For both ground and excited state potential energy surfaces, CASSCF orbitals optimized for the ground state were used. Time-dependent DFT (TD-DFT) calculations used to generate potential energy surfaces for formaldehyde employed the ωB97X-D functional^137^ because according to recent benchmarks, ωB97X-D is one of the best functionals in general for TD-DFT calculations on small organic molecules.^35^ Furthermore, the TD-DFT calculations feature a fairly dense quadrature grid using 99 radial and 590 angular points per atom for the integration of the exchange-correlation potential.^138,139^ All CC-ISR(2), ADC(2), and EOM-CCSD calculations on N_2_ and the Quest #1 database^140^ use the aug-cc-pVTZ basis set. Calculations reported for the alkenes used the def2-TZVPP basis set, and basis set dependence in these systems was assessed using def2-SVP, def2-TZVP, and def2-QZVP basis sets.^141^ The oo-pCCD, EOM-pCCD+S, and EOM-oo-pCCD+S calculations on N_2_ used the cc-pVDZ basis. We employed the aug-cc-pVDZ basis for all calculations of the formaldehyde potential energy surface.

The TD-DFT calculations presented herein were carried out using Q-Chem v6.2.^142^ CASSCF(6,6)@NEVPT2 calculations were performed using ORCA v5.0.^143^ oo-pCCD, EOM-pCCD+S, and EOM-oo-pCCD+S calculations were performed using PyBEST v2.0.0.^144,145^ Otherwise, all calculations were performed in a locally modified version of the PySCF software package.^146−148^

## 4 Results and Discussion

### 4.1 Hubbard Model

We begin our assessment of CC-ISR(2) approaches by plotting the ground state and first singlet-excited state of a ten-site, one-dimensional Hubbard model at half-filling as a function of interaction strength (*U*/|*t*|). The Hubbard excitations include contributions from double and higher excitation components that become more important as a function of interaction strength (Table S1). While our CC-ISR(2) methods are single-reference, the shifted ISR Hamiltonian does contain a (zeroth-order) description of double-excitations, so it may be possible that CC-ISR(2) methods can qualitatively describe such collective excitations to a certain extent. Our objective here is to assess (1) when the excited state character becomes too collective to be described by our single-reference methods and (2) how much the ground-state reference amplitudes matter in such a description.

**Figure 1:**
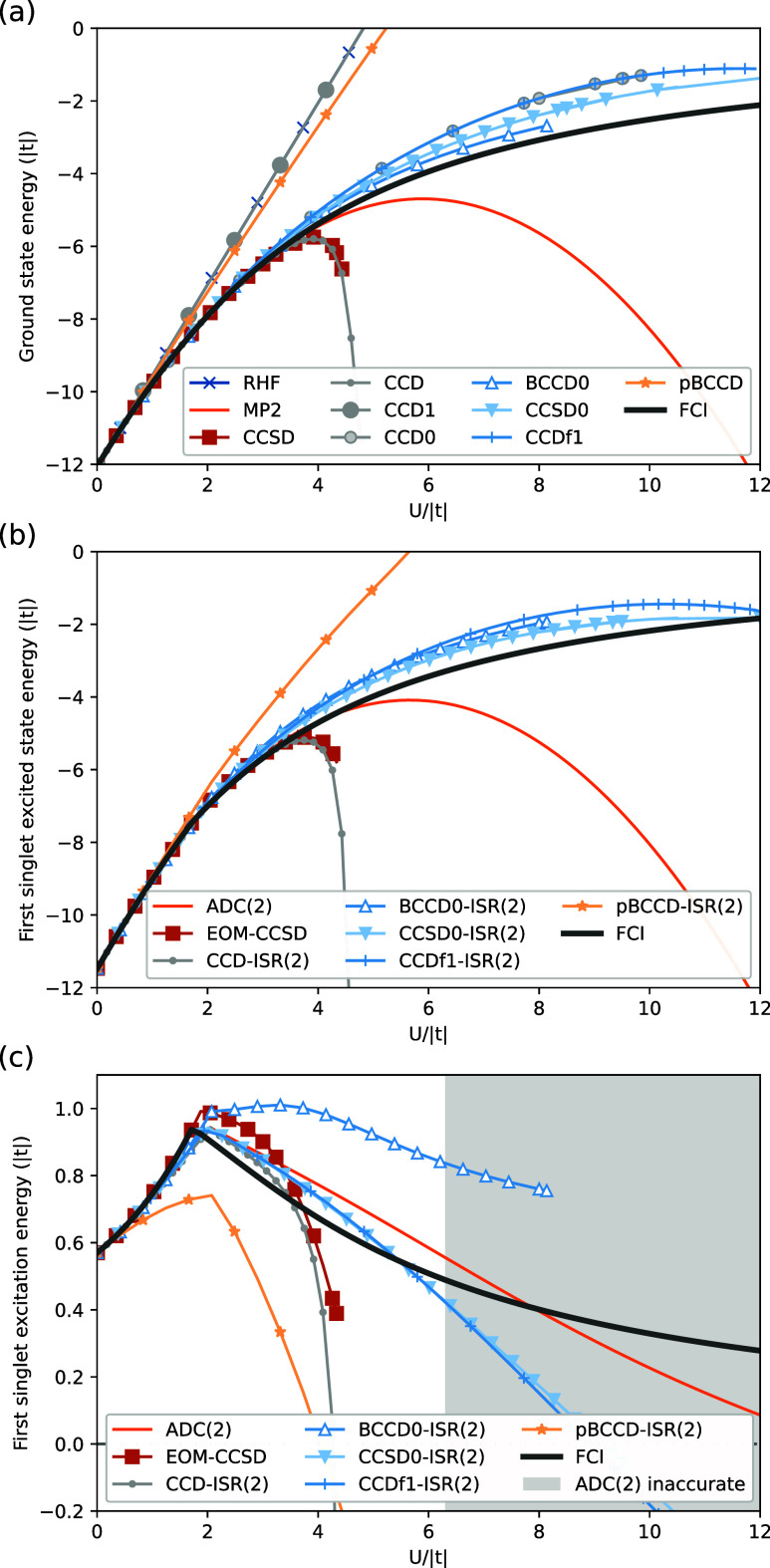
(a) Ground state (*E*_S_0__), (b) first singlet excited state (*E*_S_1__), and (c) first singlet excitation (*E*_S_1__ – *E*_S_0__) energies as a function of interaction strength (*U*/|*t* = −1.5|) for a 10-site, half-filled Hubbard model with open boundary conditions. The FCI result is exact for both states and acts as the reference. The CC/CC-ISR(2), CCSD/EOM-CCSD, and MP2/ADC(2) results are computed using canonical Hartree–Fock orbitals, and the BCC/BCC-ISR(2) results use Brueckner orbitals. The gray shaded area in (c) indicates where the MP2 energy from (a) begins to decrease, signaling divergence of the reference energy.

The results in Figure 1a are as-expected for the ground state of the Hubbard model, as each of these methods (apart from CCDf1) have been assessed before for the Hubbard model.^94,121,149,150^ The CCD and CCSD energies diverge around *U*/|*t*| = 4 while MP2 diverges slightly later, monotonically decreasing with interaction strength after *U*/|*t*| ∼ 6. With canonical Hartree–Fock (HF) or even Brueckner orbitals, pCCD fails to capture much correlation. This linear increase in the pCCD energy can be amended by using orbitals optimized with the pCCD Lagrangian (Figure S2), *localized* Brueckner orbitals, or frozen pair CCD.^94,151^ Unlike the other approaches, the CCD0, BCCD0, CCSD0, and CCDf1 ground state energies are qualitatively consistent with the exact (FCI) result. We note that the absence of off-site interactions within this Hubbard model causes CCD0 and CCDf1 to give the same result, as CCD1 adds no additional correlation energy to the HF result under these conditions. BCCD0 falls slightly closer than CCSD0 to the FCI curve, and both perform better than CCD0, suggesting that inclusion of *T̂*_1_ can be helpful for quantitative accuracy.

The lowest-energy singlet excited state of the Hubbard model is shown in Figure 1b and is qualitatively similar to the ground state case for most methods. The CCD-ISR(2) and EOM-CCSD approaches once again diverge at *U*/|*t*| ∼ 4, suggesting that they are not very well suited for cases of substantial static correlation. ADC(2) diverges in a way that is very similar to its MP2 reference, which is to be expected.

However, there are some intriguing differences in the Hubbard excited state energies. With canonical HF orbitals, pCCD-ISR(2) yields divergent Hubbard excitations closely tracking those of ADC(2), as shown in Figure S2b. We emphasize that our pCCD-ISR(2) matrix included the full singles-doubles block coupling, i.e., all double physical excitation operators were used to generate the intermediate states. In an effort to remedy the failure of canonical orbital pCCD-ISR(2), we first removed the nonpair double excitations from our ISR(2) matrix, opting instead to use only the zeroth-order pair double excitations that are parameterized in the pCCD Hamiltonian. This is equivalent to solving the pCCD-ISR(2) problem restricted to the singles configuration space, as pair double excitations do not couple to single excitations at first order. With this modification, the pCCD-ISR(2) excited state still diverged. In contrast, we found that EOM-pCCD+S (even without optimized orbitals) predicts a nondivergent Hubbard excited state, inspiring us to investigate the differences between EOM-pCCD+S and pCCD-ISR(2) more deeply.

One key difference between EOM-pCCD+S and pCCD-ISR(2) is that the former includes couplings between the reference ground state and the singly excited determinants. To study the potential impact of reference/singles coupling, we rigorously eliminate the coupling between the single excitations and the ground state by employing a pBCCD reference without localization, so that the reference MOs obey molecular point-group symmetry, akin to the conditions in our EOM-pCCD+S calculations.^94^Figure S2 shows that the resultant pBCCD-ISR(2) gives nondivergent Hubbard excitations similar to EOM-pCCD+S, emphasizing the sensitivity of methods that lack orbital invariance to orbital rotations (even when said rotations are supplied in the CI-like EOM Hamiltonian). While there is no reason to expect ISR and EOM approaches to agree quantitatively, we find it enlightening that decoupling the single excitations from the reference state leads to such similar results, essentially implicating the *T̂*_1_ contribution for the qualitative differences.

In both ground and excited states, inclusion of *T̂*_1_ in the ground state reference improves the results over those of CCD0/CCD0-ISR(2). Although BCCD0 performs better than CCSD0 in the ground state, the reverse is true in the excited state. We explain below that this is likely caused by differences in how CC and CC-based ISR(2) treat the ground state wave function along with further approximations that neglect *t*_1_ contributions in the CCSD0-ISR(2) treatment.

Overall, the methods that are most robust as the interaction strength increases are CCD0-ISR(2) and CCDf1-ISR(2), with or without the inclusion of *T̂*_1_—although their excited state energies begin (incorrectly) to decrease after *U*/|*t*| ∼ 10. Many physical systems fall within the range *U*/|*t*| ≤ 8, with *U*/|*t*| ≥ 4 considered strong correlation,^152−154^ so we anticipate rather robust treatment^152−154^ of strongly correlated systems within CCD0-ISR(2) and CCDf1-ISR(2). Interestingly, CCD0-ISR(2) performs better than EOM-CCSD0, if we judge the methods’ performance by the interaction strength at which the excited state energies begin to incorrectly turn over (Figure S3).

We can gain even more insight into the CC-ISR(2) approach by examining the excitation energies within the Hubbard model. The results in Figure 1c show the energy difference between the first singlet excited state and the ground state. The exact result exhibits a maximum near *U*/|*t*| = 2 and then decreases monotonically. The CCD-ISR(2) and EOM-CCSD approaches fail quickly with interaction strength, as suggested by the previous results. Perhaps counterintuitively, despite the divergent behavior of MP2 and ADC(2) in the absolute energies of the two states, the energy difference yields reasonable results well beyond the point at which MP2 diverges.

Despite the relatively good performance of CCD0-ISR(2), CCSD0-ISR(2), and CCDf1-ISR(2) in terms of total energies, the excitation energies begin to deviate from FCI at interaction strengths beyond *U*/|*t*| ∼ 6. We attribute this to an imbalance between the treatment of the ground state wave function in CC and the excited state wave function in the ISR(2) procedure. For example, in CCD0, the ground state wave function is written as e^*T̂*_2_^[0]^^|Φ_0_⟩, while the ground state wave function is approximated as (1 + *T̂*_2_^[0]^)|Φ_0_⟩ in the ISR(2) procedure. Whereas the CC ground state wave function and energy are calculated using the full exponential operator, thus folding in effects of quadratic contributions *T̂*_2_^2^, the excited state ISR(2) wave function is built by applying physical excitation operators to a first-order approximation to the exact ground state wave function. ADC(2) does not experience such an imbalance, as the ground- and excited state wave functions are both treated to first order; so while they both diverge, they do so at similar rates, such that the energy differences remain misleadingly reasonable. In the case of CCD0-ISR(2), the different treatment of the ground- and excited state wave functions results in an overstabilization of the excited state as the interaction parameter becomes very large.

Unlike CCSD0-ISR(2), BCCD0-ISR(2) overestimates absolute excitation energies. The Brueckner mean field determinant produces a CC wave function with rigorously zero contribution from *T̂*_1_, so that the energy of the ISR(2) ground state wave function (1 + *T̂*_2_)|Φ_0_⟩ is less underestimated. Combined with the fact that the BCCD0 energy is lower than that of CCSD0, we obtain BCCD0-ISR(2) excitation energies that are much larger than those of CCSD0-ISR(2). Surprisingly, despite the significant overestimation of excitation energies, BCCD0-ISR(2) appears to qualitatively improve the asymptotic behavior of the excitation energy surface at larger interaction strengths. This implies that using Brueckner orbitals instead of canonical orbitals may be better suited for the qualitative description of static correlation in excitation energy differences.

While excitation energies are certainly the most common metric for evaluating the performance of excited state methods, our results from the Hubbard model suggest that total energies should also be considered as important measures of a method’s accuracy. This is especially important in the case of ADC(2), where the excitation energies are qualitatively reasonable, but the forces (−∇*V*) in the ground and excited states are surely incorrect. Beyond energetic comparisons, correlation spectra and orbital-pair mutual information diagrams would be helpful to further discern which methods better capture the actual Hubbard model physics, but we leave this deeper analysis to future work.^155,156^

### 4.2 N_2_ Dissociation

**Figure 2:**
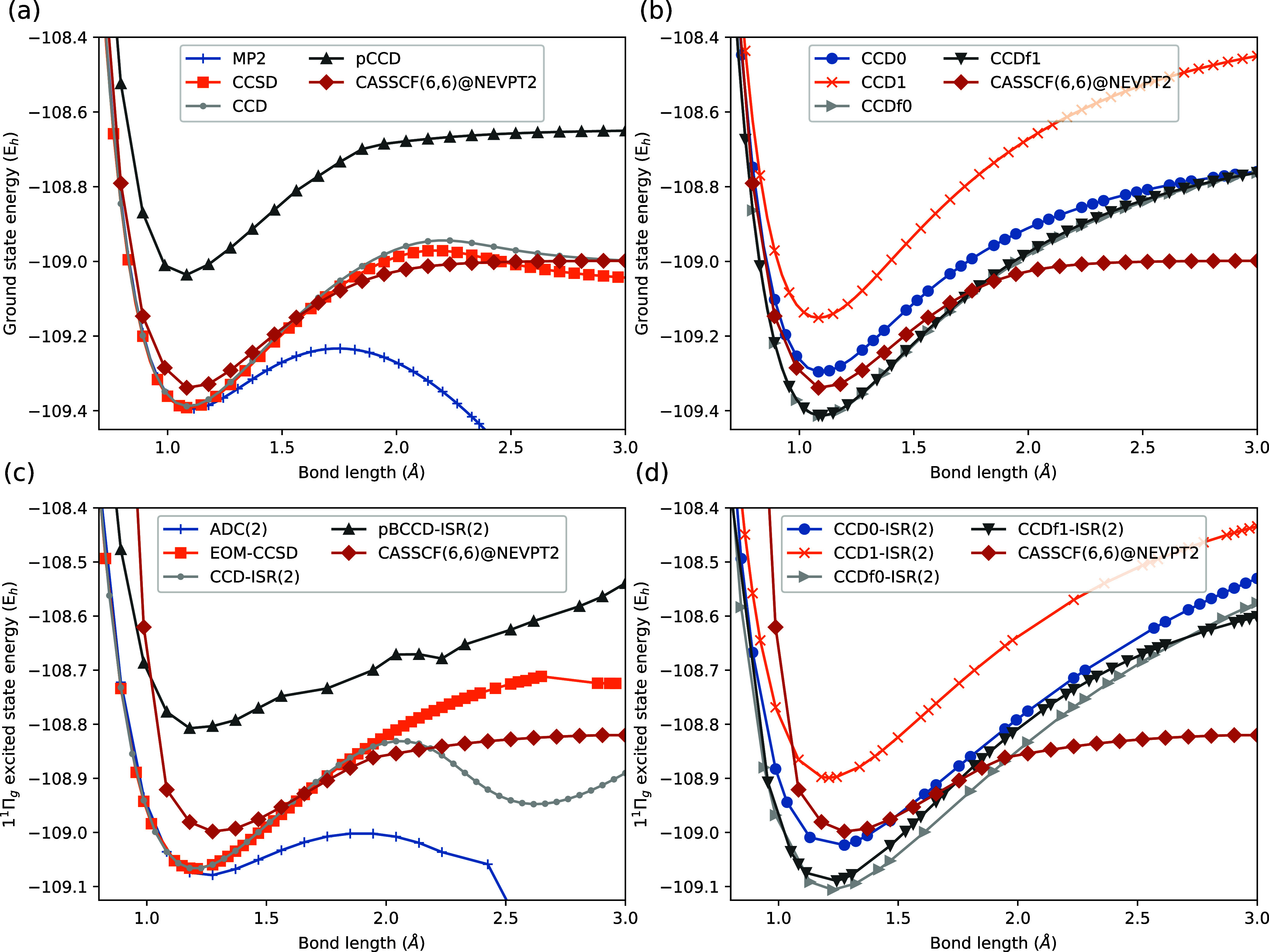
Potential energy surfaces along the bond-stretching coordinate of N_2_ molecule in (a, b) the ground state and (c, d) first degenerate singlet state, corresponding to a π → π* transition. At *R*_NN_ = 0.9 Å and 1.0 Å, the CASSCF(6,6)@NEVPT2 reference became unstable, so these two points are approximated by CASSCF alone without NEVPT2 corrections.

Moving toward a particularly challenging physical system, we now analyze the performance of various excited state approaches in the dissociation of N_2_. The results in Figure 2a,2b show the ground state potential energy surface along the N≡N bond stretch coordinate for which the restricted HF solution obeys N_2_ spatial symmetry.^157^ Throughout this section, we take the benchmark to be CASSCF(6,6)@NEVPT2 (with 6 active electrons and 6 active orbitals) for both the ground- and excited-state potential energy surfaces.

Again we find that the ground state results are predictable, with MP2 diverging and CCD and CCSD methods featuring a qualitatively incorrect barrier in the potential surface around 2 Å before the energy lowers toward the dissociation limit. Despite being much too high in energy, pCCD even with canonical orbitals performs remarkably well in capturing the qualitative shape of the potential surface relative to CASSCF(6,6)@NEVPT2. Singlet-paired CCD0 and triplet-paired CCD1 smoothly dissociate N_2_ without any artificial barriers, but the correlation energy at the equilibrium geometry is underestimated due to a lack of dynamical correlation. The equilibrium energy is captured more accurately by CCDf0 and CCDf1, as the perturbative inclusion of the missing amplitudes accounts for the missing dynamic correlation in CCD0 or CCD1, without reintroducing the artificial barrier near 2 Å. In fact, CCDf0 and CCDf1 somewhat overcorrelate at the equilibrium geometry in comparison with CCD or CCSD. The equilibrium bond lengths and vibrational frequencies predicted by all of the CC methods except for pCCD and CCD1 are all fairly close to those of CCSD (Table S3), indicating that the better-performing addition-by-subtraction CC methods retain the correct shape of the ground state potential energy surface near the equilibrium geometry. In agreement with previous work, CCDf0 and CCDf1 perform similarly in the ground state for N_2_ dissociation.^130^ While they do not diverge, all addition-by-subtraction CCD methods overestimate the energy at dissociation. At 12 Å the rough limits achieved are −108.68 eH, −108.65 eH, and −108.63 eH, for CCD0, CCDf1, and CCDf0, respectively.

The potential surface corresponding to the 1^1^Π_*g*_ excited state of N_2_ is shown in Figure 2c,2d. This is a bound excited state that corresponds to a π → π* transition, an excitation that reduces the net bond order but is not sufficient to make the state dissociative. Once again we find that ADC(2) cannot adequately describe the excited state potential surface due to the divergence of the MP2 reference state, rendering ADC(2) useless for this problem outside the Franck–Condon region.

Despite the deficiencies of CCSD in the reference state, EOM-CCSD appears to mostly smoothly dissociate N_2_ in the excited state, suggesting that the EOM procedure may iron out wrinkles in the excited state surface. Unlike EOM-CCSD, the artificial barrier in the CCD ground state is amplified in the CCD-ISR(2) excited state, which appears to be consistent with previous studies of the approximate CC-ADC(2) approach.^116^

With canonical HF orbitals, pCCD-ISR(2) predicted the N_2_ excited state to be basically unbound, as shown in Figure S4. To the contrary, we found that EOM-pCCD+S (even without optimized orbitals) predicts a bound excited state. Again suspecting the importance of *T̂*_1_ for pCCD-based excited states, we calculated pBCCD-ISR(2) and pCCSD-ISR(2) excited states, finding that only the former provided a definitively bound excited state. This supports our hypothesis that the ground/singly excited state coupling, which is not properly incorporated in CC-ISR(2) approaches that use canonical orbitals but is formally disposed of in the Brueckner basis, is likely essential in modeling the excited state of N_2_ with pCCD-based methods. We note that pCCD provides a less desirable scaffold upon which to build a proper excited-state theory, as unlike all the other CC/CC-ISR(2) methods explored in this work, pCCD (and hence also pCCD-ISR(2) and EOM-pCCD+S) is not size-consistent without orbital optimization.^97,99,100^

Similarly to their ground states, the ISR(2) approaches based on CCD0, CCD1, CCDf0, and CCDf1 references yield smooth excited state surfaces with no artificial barriers. Like in the ground state, CCDf0-ISR(2) and CCDf1-ISR(2) achieved a dissociation limit (−108.59 eH and −108.60 eH, respectively) slightly above that of CCD0-ISR(2) (−108.63 eH) at 12 Å. At the equilibrium geometry, CCDf0-ISR(2) and CCDf1-ISR(2) overcorrelate the excited state, as they did for the ground state, while CCDf1-ISR(2) overcorrelates to a lesser extent. In Figures S5 and S6, we examine the importance of the inclusion of *T̂*_1_ in the CC wave function for ISR(2) excited states based on CCD0 and CCDf1. At equilibrium, all of the approaches provide ground and excited states of similar quality. Like in the Hubbard case, CC with Brueckner orbitals leads to a higher-energy excited state than CC with single excitations.

We further quantify all of our observations by plotting the nonparallelity error (NPE) for the ground and excited states in Figure S7 with respect to the CASSCF(6,6)@NEVPT2 reference curves. Apart from supporting what we have already stated, the NPE plots reveal that, despite a distinct lack of dynamical correlation that results in a large offset from the reference energy, pCCD with Brueckner orbitals appears to capture the overall curvature of the N_2_ potential surfaces quite well. Nonetheless, without the specific choice of Brueckner orbitals, pCCD fails dramatically in the excited state.

Overall, the approaches with smooth ground and ^1^Π_*g*_ potential energy surfaces without divergence or anomalous barriers are CCD0/CCD0-ISR(2), CCD1/CCD1-ISR(2), CCDf0/CCDf0-ISR(2), and CCDf1/CCDf1-ISR(2), their counterparts with the inclusion of *T̂*_1_ in some fashion, and pBCCD/pBCCD-ISR(2). Our results suggest that improving the qualitative nature of the reference wave function can improve excited state descriptions within an ISR framework.

### 4.3 Formaldehyde PES Topology

Next, we emphasize some of the more favorable attributes of ISR methods. Namely, the Hermitian ISR framework has the capacity to circumvent several major problems with the EOM-CC approach. While we will not focus on this here, one consequence of the non-Hermitian framework of EOM-CC is that the predicted intensities are not size-intensive.^158,159^ Thus, intensities of local excitations on a chromophore can be perturbed by the presence of an atom that is infinitely far away (yet present) in the calculation. Notably, linear response CC is an alternative to EOM-CC that, while still non-Hermitian, provides size-intensive intensities, but being that EOM-CC is more commonly used, we focus on EOM-CC models here.^103^

Another problem with the non-Hermitian formulation of EOM-CC is that conical intersections/avoided crossings between two excited states of the same symmetry are not correctly described. The non-Hermitian EOM-CC Hamiltonian imparts a lack of orthogonality between the excited states and can lead to complex solutions to the EOM problem. On the other hand, the CC-ISR(2) approach imposes orthogonality between the excited states, and the Hamiltonian is Hermitian in the correlated excited state basis. In this sense, CC-ISR(2) methods should correctly describe the topology of conical intersections/avoided crossings between excited states of the same symmetry and predict size-intensive oscillator strengths. Note that the conical intersection problem between excited states is distinct from correctly predicting S0/S1 conical intersection topology between ground and excited states. At the moment, no ISR(2)-based method (apart from the aforementioned spin-flip variants) can correctly predict S0/S1 conical intersection topology due to an imbalance in the treatment of ground and excited states;^160^ a problem shared with TD-DFT.

**Figure 3:**
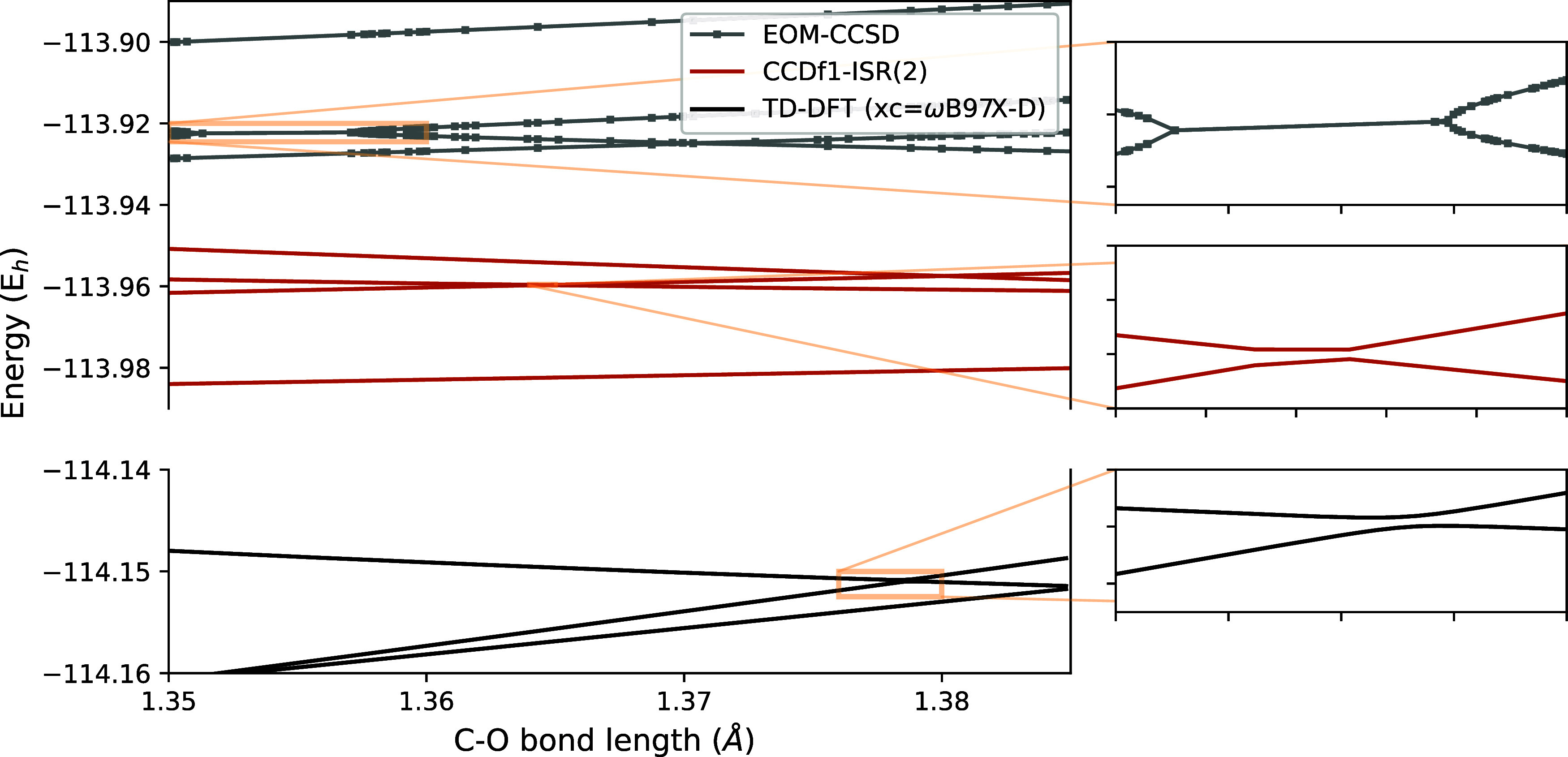
Excited state energies for formaldehyde as a function of C=O bond length. We used the α_OCH_ = 118°, *R*_CH_ = 111.915 pm geometry specified in ref (109). The S5–S6 (2 ^1^A_1_-3 ^1^A_1_) avoided crossing predicted by TD-DFT and CCDf1-ISR(2) and incorrect degeneracy region predicted by EOM-CCSD are shown in the insets.

We assess the ability of CC-ISR(2) to predict potential energy surface topology of two isosymmetric excited states by examining the notorious case of C=O bond stretching in formaldehyde.^109^ Although similarity-constrained EOM-CCSD can predict an avoided crossing,^110,161^ our standard EOM-CCSD results in Figure 3 exhibit the expected incorrect topology. Because TD-DFT correctly captures the topology of isosymmetric conical intersections between two excited states, we have included it as a qualitative metric. Our TD-DFT calculations reveal that the 2 ^1^A_1_-3 ^1^A_1_ surfaces do indeed undergo an avoided crossing, which is qualitatively consistent with our CCDf1-ISR(2) result. Considering that conical intersection topology can have a qualitative impact on photodynamics, this is a clear advantage of CCDf1-ISR(2) over EOM-CC approaches.^162^

### 4.4 Performance on Quest#1 Database

**Figure 4:**
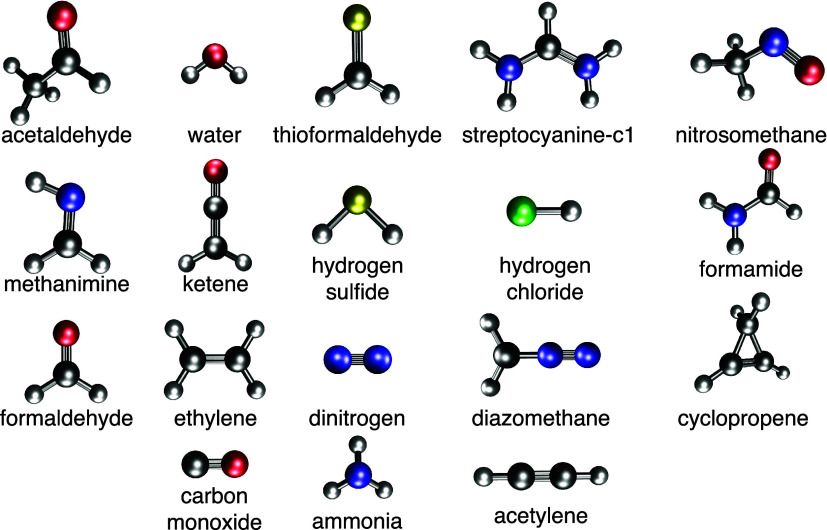
18 molecules in the Quest #1 database.^140^ We performed calculations on the 17 closed-shell molecules in this set, which excludes one open-shell molecule (streptocyanine-c1).

**Figure 5:**
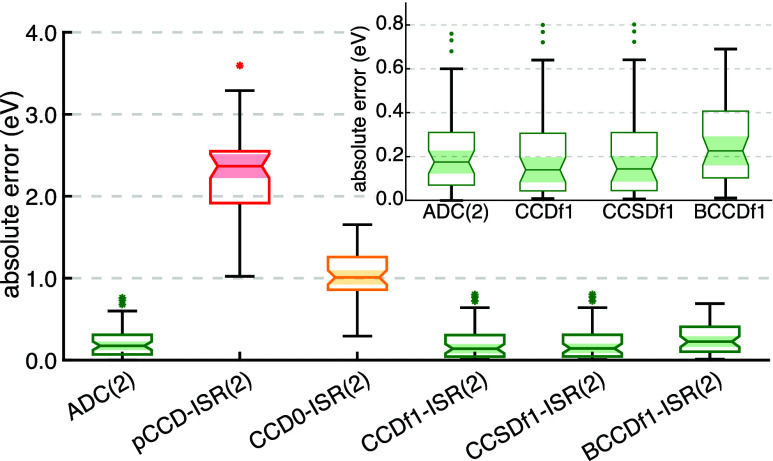
Error statistics (in eV) of 52 singlet excitation energies for the 17 closed-shell molecules in the Quest #1 database.^140^ Excitation energies are compared with the aug-cc-pVTZ basis Quest #1 theoretical best estimates. Upper and lower delimiters indicate the maximum and minimum error, while the upper and lower bounds of each box indicate the upper and lower quartiles, respectively. Median absolute errors are indicated by the central horizontal line and outliers by asterisks. Overlapping notches (highlighted by shaded regions) indicate a statistical similarity between distributions up to a 95% confidence interval. The ADC(2) data was taken from the Quest #1 database. The inset shows the error distributions for ADC(2) and the CC-ISR(2) approaches with lowest error in more detail.

Thus, far we have expounded upon the capacity for CCDf1-ISR(2) to correctly describe potential energy surface topology and topography with little mention of the quality of CCDf1-ISR(2) absolute excitation energies. Our focus on potential surfaces was motivated by the fact that nonadiabatic photodynamics simulations depend more strongly on the shapes of the potential surfaces (i.e., derivatives, forces) than they do on the absolute energies, but accurate energy differences at the equilibrium geometry are also necessary. To this end, we benchmarked our suite of CC-ISR(2) approaches on the QUEST #1 organic molecules data set of Loos et al.,^140^ pictured in Figure 4. The results in Figure 5 emphasize the importance of proper inclusion of dynamical correlation effects to the computed excitation energy, as our CC-ISR approaches exhibit mean absolute errors (MAE) that decrease monotonically with greater inclusion of dynamical correlation. Specifically, we find MAE of 2.3, 1.0, and 0.21 eV for pCCD-ISR(2), CCD0-ISR(2), and CCDf1-ISR(2), respectively. We note that pCCD-ISR(2) and EOM-pCCD+S even with canonical orbitals can incorrectly break degeneracies (Table S4). We excluded five pCCD-ISR(2) excitation energies (for CO and N_2_) because the predicted degeneracy pattern was incorrect. Our BCCDf1-ISR(2) approach (MAE 0.26 eV) is comparable in accuracy to ADC(2) (MAE 0.23 eV), which is consistent with the results reported in Table 3 of ref (113) for CCD-ISR(2).

We note that CCDf0-ISR(2) gives a slightly larger MAE of 0.28 eV. This is likely because the triplet amplitudes represent a smaller fraction of the total correlation energy beyond HF, so solving for those first, then freezing them and computing the infinite-order solution to eq 14 ensures that the largest component of the correlation energy is iteratively optimized with at least approximate external amplitudes provided by the triplet contribution. Solution of the CCDf0 equations imparts more error by approximating the larger (singlet) contribution in the absence of other amplitudes while the infinite-order external CC equation for the remaining triplet component only offers small corrections.

Including *T̂*_1_ through CCSDf1-ISR(2) did not affect the quality of the computed Quest #1 excitation energies, giving a MAE of 0.21 eV. While BCCDf1-ISR(2) gave a slightly increased MAE of 0.26 eV, the error margins are statistically the same as ADC(2) and other approaches, as indicated by the overlapping shaded regions in the inset of Figure 5. Interestingly, using the slightly more justified Brueckner determinant and corresponding *t*_2_ amplitudes as a starting point for ISR(2) results in the elimination of outliers in the statistics, meaning the BCCDf1-ISR(2) provides a more even-handed description across all varieties of excited state represented in Quest #1. Overall, our Quest #1 benchmarking results suggest that we can use BCCDf1-ISR(2) to attain the same level of accuracy in excitation energies as ADC(2) and full CCD-ISR(2) but with a better overall treatment of static correlation, manifesting in potential energy surface topographies that are significantly more accurate than those provided by either of the latter approaches.

### 4.5 Double Excitations

Quantitatively accurate models of excitations with substantial double excitation character generally require wave function methods containing at least triple substitutions, such as a multireference CAS-based method or ADC(3). While our CC-ISR(2) approaches do not contain triple excitations, they do model double excitations to zeroth order in the energy, permitting at least some coverage of the doubles manifold. To understand the extent to which CC-ISR(2) approaches can account for double excitations, we assessed the performance of CCSDf1-ISR(2) for several linear polyenes. Such trans-polyenes host ^1^A_*g*_ (π → π*) states that are known to have substantial double-excitation character. Table 1 shows excitation energies for ethene, butadiene, hexatriene, and octatetraene, as calculated with the Def2-TZVPP basis set. As expected, CCSDf1-ISR(2) performs slightly better than ADC(2) (by about 0.1 eV) even for double excitations and seems to be on par with EOM-CCSD for the ^1^*A*_*g*_ states while outperforming EOM-CCSD for the ^1^*B*_1*u*_ states. These results suggest that improving the ground-state reference can impart improvements to the predicted excitation energies of transitions that have pronounced double-excitation character.

**Table 1:** Excitation Energies (eV) for Four Alkenes

system	state	EOM-CCSD	ADC(2)	CCSDf1-ISR(2)	TBE^a^
C_2_H_4_	1 ^1^B_1*u*_	8.31	8.21	8.11	7.80
C_4_H_6_	1 ^1^B_1*u*_	6.55	6.28	6.17	6.18
	2 ^1^A_*g*_	7.45	7.59	7.48	6.55
C_6_H_8_	1 ^1^B_1*u*_	5.55	5.22	5.09	5.10
	2 ^1^A_*g*_	6.66	6.65	6.54	5.09
C_8_H_10_	2 ^1^A_*g*_	6.02	5.87	5.74	4.47
	1 ^1^B_1*u*_	4.91	4.53	4.40	4.66
Mean Absolute Error					
	1 ^1^B_1*u*_	0.40	0.19	0.15	
	2 ^1^A_*g*_	1.34	1.33	1.22	
	All	0.80	0.68	0.61	

a Theoretical best estimates from ref (163).

## 5 Conclusions and Outlook

We have introduced addition-by-subtraction coupled cluster (CC) with double substitutions (CCD) approaches as an *ansatz* for the intermediate state representation (ISR) approach for calculating excited states in order to account for nondynamical correlation with polynomial-scaling, single-determinant reference wave functions. Among those tested were pair CCD (pCCD), singlet-paired CCD (CCD0), triplet-paired CCD (CCD1), and CCD with frozen singlet/triplet-paired amplitudes (CCDf0/CCDf1), as well as these methods with CC-singles excitations or Brueckner instead of canonical HF orbitals.

Of course, there will always be limitations in the single-reference description of static correlation,^164,165^ but all CC-ISR(2) approaches except for pCCD-ISR(2) with canonical orbitals were capable of correctly predicting the topography of excited state potential energy surfaces along bond dissociation coordinates with qualitative accuracy. For quantitative accuracy of excitation energies and qualitatively accurate potential surfaces, we recommend BCCDf1-ISR(2), as it captures a greater fraction of dynamical correlation and predicts excitation energies with statistical accuracy on par with that of second-order algebraic diagrammatic construction [ADC(2)] but without the dramatic failures often exhibited by ADC(2) when the underlying second-order Møller–Plesset (MP2) reference energy diverges. Importantly, BCCDf1-ISR(2) is somewhat more formally sound than its CCDf1-ISR(2) or CCSDf1-ISR(2) counterparts, as it rigorously eliminates singles/reference-state coupling that should otherwise appear at each order in the ISR equations.

The Hermitian construction of the ISR excited states also allows CCDf1-ISR(2) to describe avoided crossings and conical intersections correctly where equation-of-motion (EOM) CC methods may fail, albeit at a slight sacrifice of accuracy in the excitation energies. While CCDf1-ISR(2) itself may not enter widespread use, we believe that future adaptations of the approach will be useful for modeling photodynamics due to their capacity to correctly describe potential energy surface topology and topography.

Some of the most important contributions of this work are conceptual advancements that were made in our first steps toward finding optimal single-reference excited state theories. First, that some—but not all—reference wave functions that provide qualitatively good descriptions of statically correlated systems in their electronic ground state can also improve the quality of the predicted excited states. Especially important is the remarkable failure of pCCD-ISR(2) in the dissociation of N_2_, where the ground state pCCD topography was perhaps among the best of the methods that we tested. While this may not necessarily be the case for EOM- or linear response-based approaches, within the ISR formulation, pCCD reference wave functions with canonical HF MOs do not even qualitatively capture static correlation in excited states. Although these failures can be somewhat alleviated by choosing the Brueckner orbital basis, our results emphasize that caution should be exercised when calculating excited states with methods that are not invariant to unitary transformations of the orbital basis within the occupied or virtual spaces. Our results warrant more extensive studies into the impacts of using non-orbital-invariant methods to model optical spectra of molecules, especially as it pertains to subtle changes in symmetry that manifest in complex chemical environments.

We also demonstrated the usefulness of the ISR procedure in improving the description of an avoided crossing. This motivates our further study into a more formal derivation of an ISR framework from a variety of CC reference wave functions employing a Hermitian framework. A more rigorous approach than CC-ISR(2) should treat the ground and excited state wave function at the same level of approximation. (Preliminary testing suggests that this may be necessary for the quantitative treatment of absolute excitation energies in transition metal-containing molecules.) Additionally, we are actively investigating related CC approaches that may avoid the  scaling of CCDf1-ISR(2), which is bottlenecked by the solution of the CCDf1 amplitudes. With more concrete formalism and lower computational cost, methods similar to CCDf1-ISR(2) may offer efficient yet robust alternatives to complete active-space approaches to calculating photodynamics of strongly correlated excited states that typify common processes such as photolysis. Most importantly, it would seem that the general concept of optimal single-reference theories for excited states (the idea that improvements in the initial reference wave function can beget improvements to the predicted excited states) is well-founded and merits further research.

## Supporting Information Available

Numerical N_2_ PES data (XLSX)
Hubbard model FCI determinant weights (Table S1); additional Hubbard model plots (Figures S1–S3); additional PES curves for N_2_ (Figures S4–S6); nonparallelity errors for N_2_ (Figure S7); comparison of N_2_ PESs between TZ and QZ bases (Figure S8); basis set dependence of alkene excitation energies (Table S2, Figures S9 and S10); ground state N_2_ spectroscopic parameters (Table S3); and two diatomics’ excitation energies from pCCD-based methods (Table S4) (PDF)
