# Supplementary material for: Optimal-Reference Excited State Methods: Static Correlation at Polynomial Cost with Single-Reference Coupled-Cluster Approaches
Source: J Chem Theory Comput. 2025 Apr 1;21(8):4080–94. doi: 10.1021/acs.jctc.5c00172 (PMC12020369; doi:10.1021/acs.jctc.5c00172)
Supplement: Supplementary file 2 — ct5c00172_si_002.pdf [file ct5c00172_si_002.pdf]

Supporting Information for
Optimal-Reference Excited State Methods: Static Correlation at Polynomial Cost with Single-Reference Coupled-Cluster Approaches

Sylvia J. Bintrim and Kevin Carter-Fenk*
Department of Chemistry, University of Pittsburgh, Pittsburgh, Pennsylvania 15218, USA
(Dated: March 24, 2025)

*kay.carter-fenk@pitt.edu

1. Excited state character in the Hubbard model

One might ask whether the 'kink' in the excitation energy plot near $U/|t| = 2$ indicates the presence of a higher-energy singlet excitation with the same orbital character as the excited state curve we started on at low U. We show that this is not the case. As can be seen in Table S1, the main determinants (and their coefficients' signs) contributing to the S0 ground and S1 state remain the same as a function of interaction strength from $U/|t| = 1$ to $U/|t| = 3$. The ground state RHF MO coefficients for the HOMO, LUMO, and LUMO+1 are independent of interaction strength. Because the ground and excited state characters as well as the MO energetic ordering are independent of $U/|t|$ across the 'kink' in the excitation energy around $U/|t| = 2$, we can conclude that we are, in fact, tracking the same first-singlet excited state as a function of interaction strength.

There is a notable enhancement of collective character in the ground and excited states even at interaction strengths as low as $U/|t| = 3$. So, while we are confident in our state assignment for the lowest-energy excited state (the ordering of states does not change with interaction strength), we note that the excited states of the Hubbard model provide an exacting test for how well a method can deal with collective excitation character. All of the methods that we test in the main text can capture at least *some* plasmonic character, as they explicitly account for double-excitations.

In Figure S1, we show that even CIS can predict the kink in the excitation energy plot, though it occurs at much higher $U/|t|$ than predicted by other methods due to the lack of inclusion of doubles-and-higher substitutions in CIS.

Table S1. Configuration Interaction Coefficients for the Hubbard Model as a Function of Interaction Strength

Ground state

$\|U/t\|$	Dominant Configurations	C	$\|C^2\|$
1.0	$ijklm, \bar{i}\bar{j}\bar{k}\bar{l}\bar{m}$	0.969	0.939
	$ijkla, \bar{i}\bar{j}\bar{k}\bar{l}\bar{a}$ (double)	−0.116	0.014
3.0	$ijklm, \bar{i}\bar{j}\bar{k}\bar{l}\bar{m}$	0.754	0.568
	$ijkla, \bar{i}\bar{j}\bar{k}\bar{l}\bar{a}$ (double)	−0.256	0.066
	$ijkma, \bar{i}\bar{j}\bar{k}\bar{l}\bar{b}$	0.132	0.017
	$ijklb, \bar{i}\bar{j}\bar{k}\bar{m}\bar{a}$	0.132	0.017
20	$ijklm, \bar{i}\bar{j}\bar{k}\bar{l}\bar{m}$		0.038
	$ijkla, \bar{i}\bar{j}\bar{k}\bar{l}\bar{a}$ (double)		0.014
	$ijklb, \bar{i}\bar{j}\bar{k}\bar{m}\bar{a}$		0.008

First singlet excited state

$\|U/t\|$	Dominant Configurations	C	$\|C^2\|$
1.0	$ijklm, \bar{i}\bar{j}\bar{k}\bar{l}\bar{a}$ (single)	−0.691	0.478
	$ijkla, \bar{i}\bar{j}\bar{k}\bar{l}\bar{m}$ (single)	−0.691	0.478
	$ijklm, \bar{i}\bar{j}\bar{k}\bar{l}\bar{b}$ (single)	−0.051	0.003
	$ijklb, \bar{i}\bar{j}\bar{k}\bar{l}\bar{m}$ (single)	−0.051	0.003
3.0	$ijklm, \bar{i}\bar{j}\bar{k}\bar{l}\bar{a}$ (single)	−0.603	0.364
	$ijkla, \bar{i}\bar{j}\bar{k}\bar{l}\bar{m}$ (single)	−0.603	0.364
	$ijklm, \bar{i}\bar{j}\bar{k}\bar{l}\bar{b}$ (single)	−0.105	0.011
	$ijklb, \bar{i}\bar{j}\bar{k}\bar{l}\bar{m}$ (single)	−0.105	0.011
20	$ijkla, \bar{i}\bar{j}\bar{k}\bar{l}\bar{a}$ (double)	−0.123	0.015

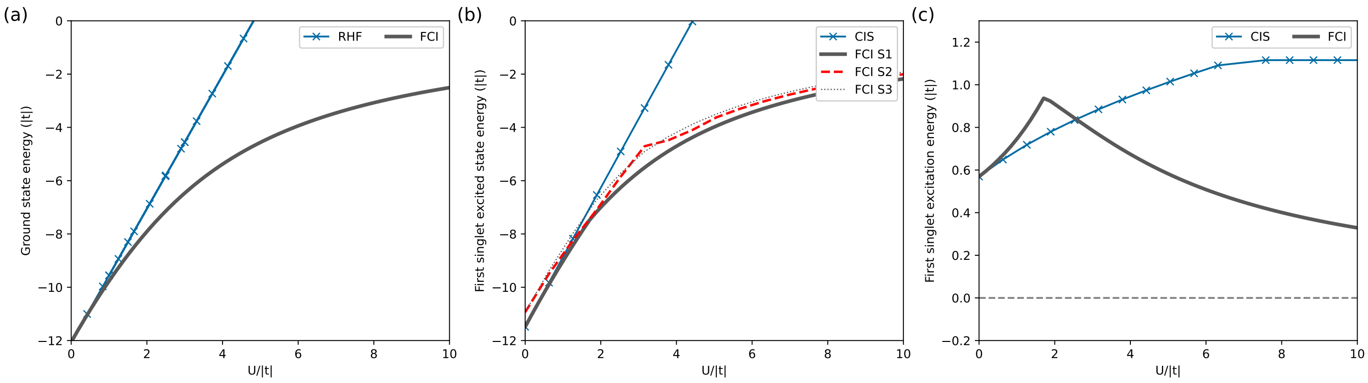

Figure S1. (a) Ground state (E_{S_0}), (b) singlet excited states, and (c) first singlet excitation ($E_{\mathrm{S}_1} - E_{\mathrm{S}_0}$) energies as a function of interaction strength ($U/|t = -1.5|$) for a 10-site, half-filled Hubbard model with open boundary conditions. The FCI result is exact for both states and acts as the reference.

2. pCCD-based methods for the Hubbard model

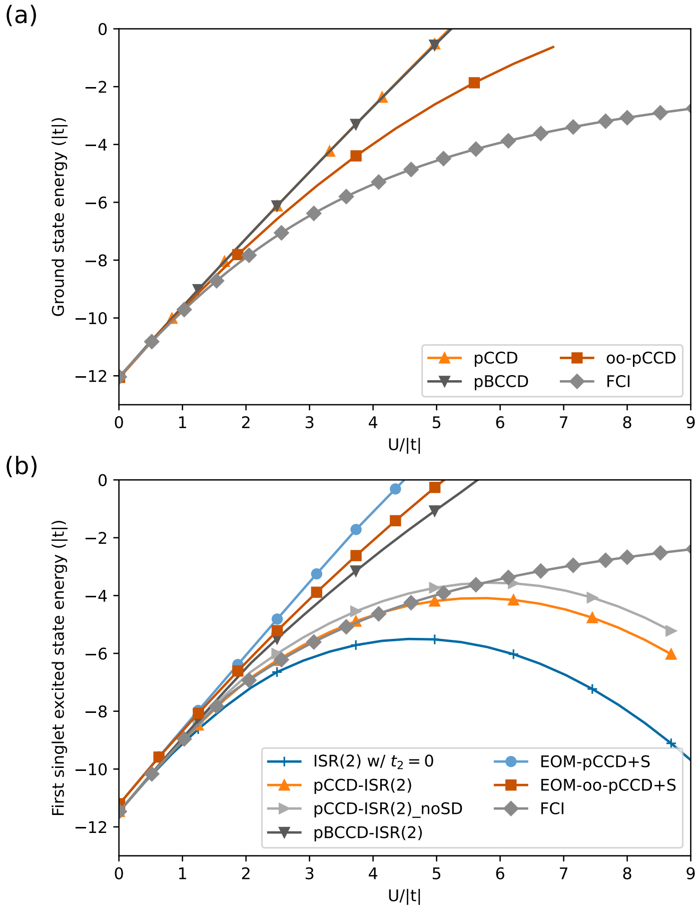

Figure S2. (a) Ground state (E_{S_0}) and (b) first singlet excited state (E_{S_1}) as a function of interaction strength $(U/|t = -1.5|)$ for a 10-site, half-filled Hubbard model with open boundary conditions. The FCI result is exact for both states and acts as the reference. 'noSD' denotes that singles-doubles coupling is set to zero.

3. EOM-CCSD0 for the Hubbard model

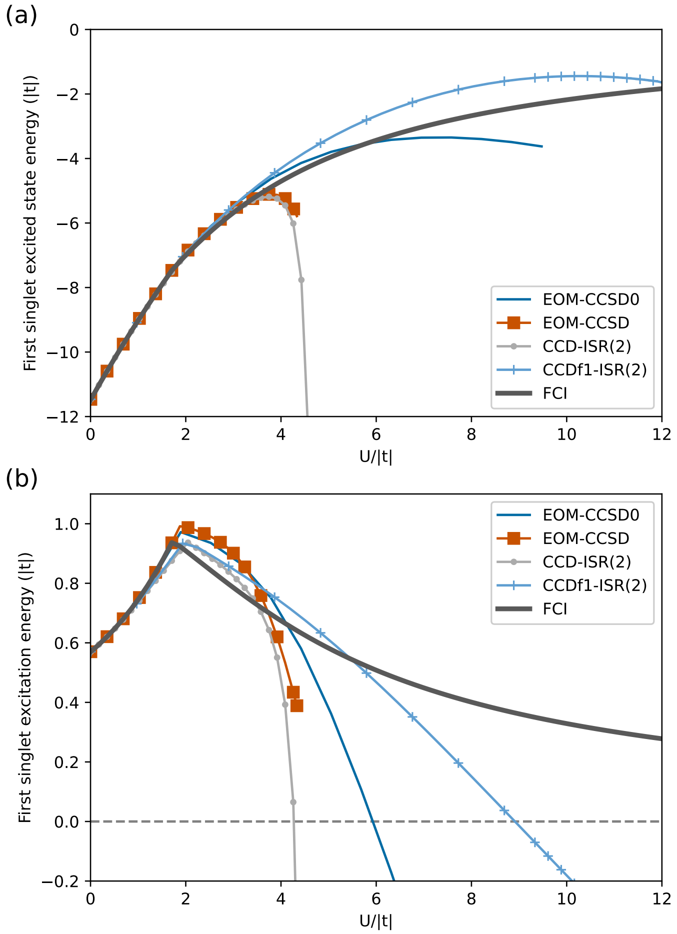

Figure S3. (a) First singlet excited state (E_{S_1}) and (b) first singlet excitation energy ($E_{\mathrm{S}_1} - E_{\mathrm{S}_0}$) as a function of interaction strength ($U/|t = -1.5|$) for a 10-site, half-filled Hubbard model with open boundary conditions. The FCI result is exact and acts as the reference.

4. pCCD-based methods for N_2 dissociation

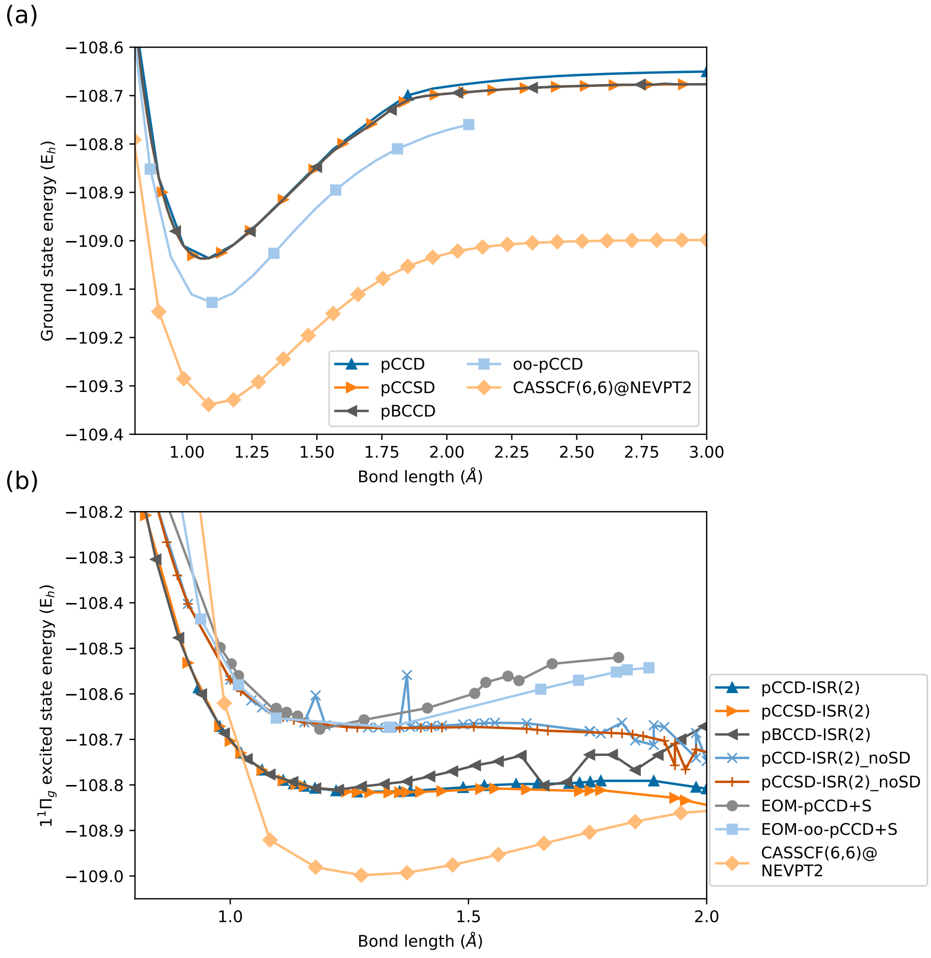

Figure S4. Potential energy surfaces along the bond-stretching coordinate of N_2 molecule in (a) the ground state and (b) first degenerate singlet state, corresponding to a $\pi \rightarrow \pi^*$ transition. 'noSD' denotes that singles-doubles coupling is set to zero.

5. CCD0/CCD0-ISR(2) with $\hat{T}_1$: choices for N_2 dissociation

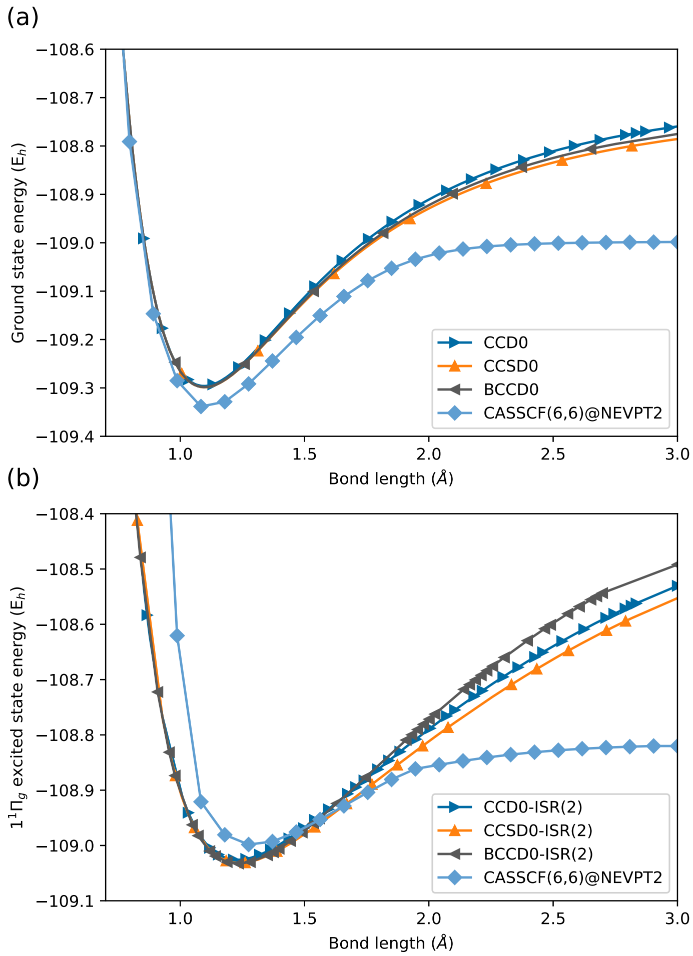

Figure S5. Potential energy surfaces along the bond-stretching coordinate of N_2 molecule in (a) the ground state and (b) first degenerate singlet state, corresponding to a $\pi \rightarrow \pi^*$ transition.

6. CCDf1/CCDf1-ISR(2) with $\hat{T}_1$: choices for N_2 dissociation

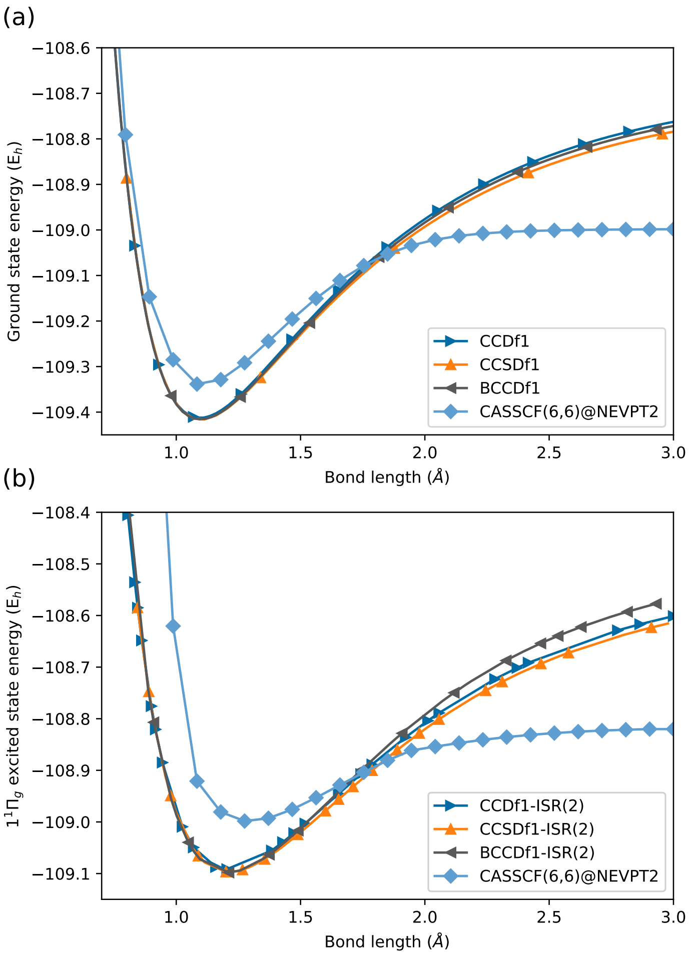

Figure S6. Potential energy surfaces along the bond-stretching coordinate of N_2 molecule in (a) the ground state and (b) first degenerate singlet state, corresponding to a $\pi \rightarrow \pi^*$ transition.

7. Non-parallelity error plots for N_2 dissociation

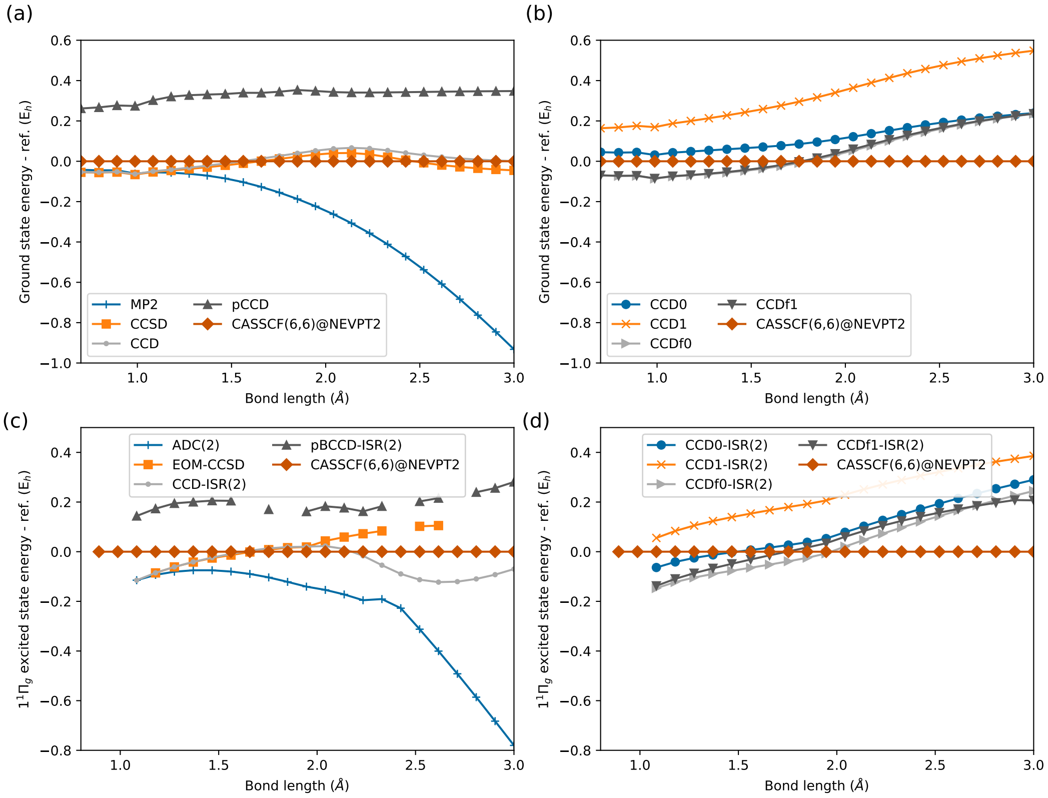

Figure S7. Deviation of potential energy surface from the reference potential energy surface (CASSCF(6,6)@NEVPT2) along the bond-stretching coordinate of N_2 molecule in (a,b) the ground state and (c,d) first degenerate singlet state, corresponding to a $\pi \rightarrow \pi^*$ transition.

8. Basis set dependence of N_2 dissociation

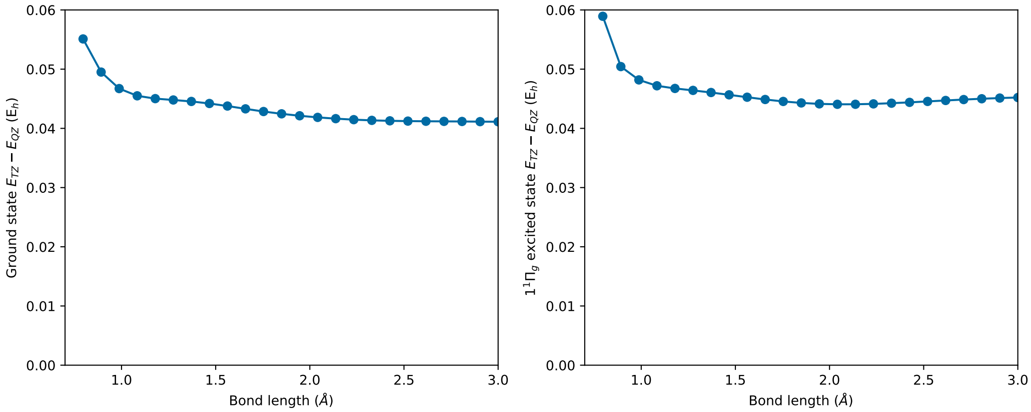

Figure S8. Deviation between CCD0/CCD0-ISR(2) potential energy surfaces computed in the aug-cc-pVTZ and aug-cc-pVQZ bases along the bond-stretching coordinate of the N_2 molecule in (a) the ground state and (b) first degenerate singlet state, corresponding to a $\pi \rightarrow \pi^*$ transition.

9. Basis set dependence of alkene excitations energies

Table S2. Excitation energies (eV) for alkenes in the def2-(SVP/TZVP/QZVP) bases

system	state	EOM-CCSD	ADC(2)	CCSDf1-ISR(2)	TBE[a]
C_2H_4	$1\ ^1B_{1u}$	8.94/8.33/8.15	8.80/8.23/8.06	8.70/8.14/7.97	7.80
C_4H_6	$1\ ^1B_{1u}$	6.97/6.56/6.44	6.66/6.30/6.19	6.53/6.19/6.09	6.18
	$2\ ^1A_g$	7.61/7.46/7.33	8.01/7.61/7.33	7.88/7.50/7.32	6.55
C_6H_8	$1\ ^1B_{1u}$	5.89/5.56/	5.50/5.23/	5.36/5.10/	5.10
	$2\ ^1A_g$	6.70/6.66/	6.89/6.66/	6.76/6.55/	5.09
C_8H_{10}	$2\ ^1A_g$	6.05/6.02/	6.06/5.87/	5.92/5.75/	4.47
	$1\ ^1B_{1u}$	5.20/4.92/	4.77/4.54/	4.62/4.40/	4.66
MAE		1.07/0.81/	0.98/0.69/	0.86/0.61/	

[a] Theoretical best estimates (TBE) from Ref. 1

Mean absolute errors (MAE) were computed with respect to the TBE.

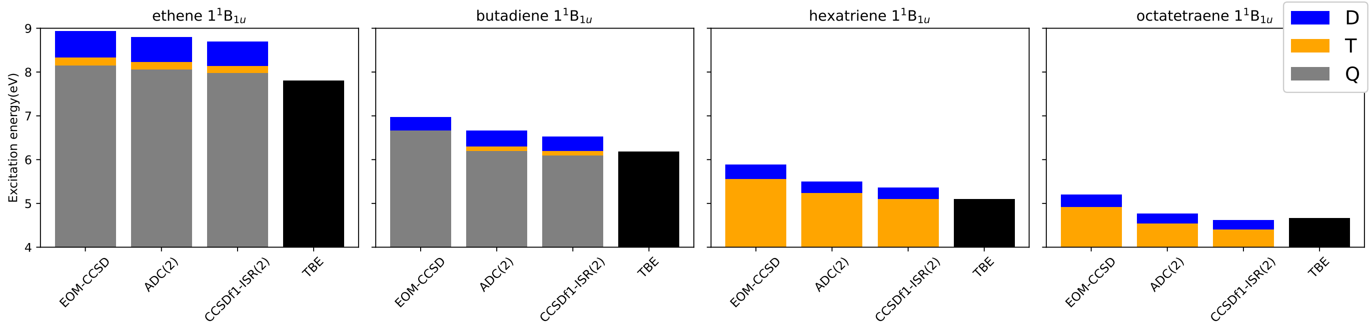

Figure S9. Basis set dependence of alkenes' 1^1B_{1u} excitation energies. TBE data from Ref. 1

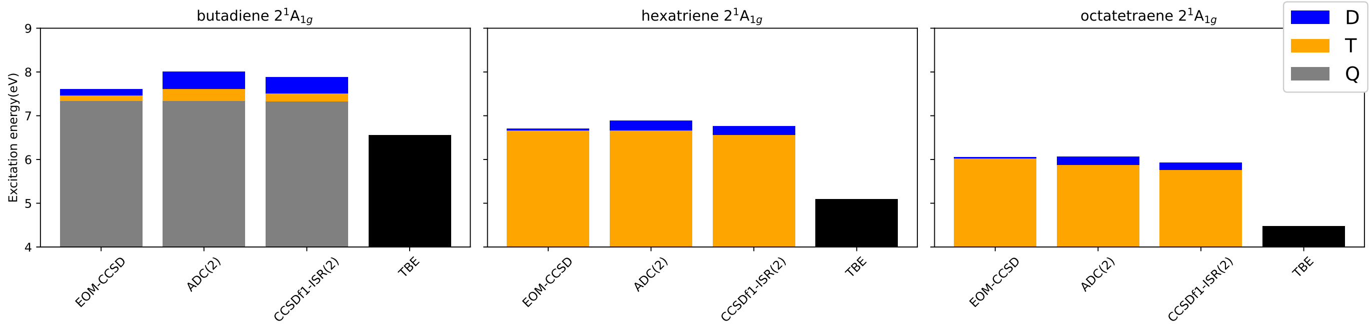

Figure S10. Basis set dependence of alkenes' 2^1A_{1g} excitation energies. TBE data from Ref. 1

10. N_2 spectroscopic parameters

Table S3. N_2 ground state equilibrium bond length (Å) and vibrational frequency (cm^{-1})

method	R_{eq}	ω
CCSD	1.093	2449
CCSDf1	1.095	2432
CCD	1.092	2460
CCDf1	1.094	2445
CCD0	1.095	2423
CCDf0	1.095	2425
CCD1	1.073	2627
pCCD	1.064	2715

For our finite difference Hessian calculation on top of PySCF's CC analytical nuclear gradients, we took a step size $\Delta r = 0.0001$Å. The atomic mass of ^{14}N was used (14.003 u).

11. Equilibrium geometry N_2 and CO excitation energies from pCCD-based methods

Table S4. N_2 and CO excitation energies (eV) in the aug-cc-pVTZ basis

system	state	ADC(2)	EOM-pCCD+S	pCCD-ISR(2)	TBE[a]
CO	$1\,^1\Sigma^+ \rightarrow 1\,^1\Pi(n \rightarrow \pi^\star)$	8.68	9.355	6.358	8.49
	$1\,^1\Sigma^+ \rightarrow 1\,^1\Sigma^-(\pi \rightarrow \pi^\star)$	10.02	9.922	6.884	9.92
	$1\,^1\Sigma^+ \rightarrow 1\,^1\Delta(\pi \rightarrow \pi^\star)$	10.30	9.943/10.354	7.151/7.298	10.06
	$1\,^1\Sigma^+ \rightarrow 2\,^1\Sigma^+$	11.33	12.299	9.896	10.95
	$1\,^1\Sigma^+ \rightarrow 3\,^1\Sigma^+$	11.84	12.866	10.497	11.52
	$1\,^1\Sigma^+ \rightarrow 2\,^1\Pi$	12.04	13.097	10.671	11.72
N_2	$1\,^1\Sigma_g \rightarrow 1\,^1\Pi_g(n \rightarrow \pi^\star)$	9.47	9.558/9.647	6.743	9.34
	$1\,^1\Sigma_g \rightarrow 1\,^1\Sigma_u^-(\pi \rightarrow \pi^\star)$	10.25	9.000	6.720	9.88
	$1\,^1\Sigma_g \rightarrow 1\,^1\Delta_u(\pi \rightarrow \pi^\star)$	10.79	10.399	7.221/7.489	10.29
	$1\,^1\Sigma_g \rightarrow 1\,^1\Sigma_g^+$	13.01	–	11.480	12.98
	$1\,^1\Sigma_g \rightarrow 1\,^1\Pi_u$	13.34	–	–	13.03
	$1\,^1\Sigma_g \rightarrow 1\,^1\Sigma_u^+$	13.08	–	–	13.09
	$1\,^1\Sigma_g \rightarrow 2\,^1\Pi_u$	13.95	–	–	13.46

[a]Theoretical best estimates (TBE) and geometries from Ref. 2

References

(1) Schreiber, M.; Silva-Junior, M. R.; Sauer, S. P. A.; Thiel, W. Benchmarks for electronically excited states: CASPT2, CC2, CCSD, and CC3. *J. Chem. Phys.* **2008,** *128*, 134110:1–25.

(2) Loos, P.-F.; Scemama, A.; Blondel, A.; Garniron, Y.; Caffarel, M.; Jacquemin, D. A mountaineering strategy to excited states: Highly accurate reference energies and benchmarks. *J. Chem. Theory Comput.* **2018,** *14,* 4360–4379.
